# Citrate anticoagulation and systemic heparin anticoagulation during continuous renal replacement therapy among critically-ill children

**DOI:** 10.1038/s41390-024-03163-x

**Published:** 2024-03-30

**Authors:** Seyma Koksal Atis, Muhterem Duyu, Zeynep Karakaya, Alev Yilmaz

**Affiliations:** 1 Department of Pediatrics, Istanbul Goztepe Prof. Dr. Suleyman Yalcin City Hospital, Istanbul, Turkey; 2 Pediatric Intensive Care Unit, Istanbul Goztepe Prof. Dr. Suleyman Yalcin City Hospital, Istanbul, Turkey; 3 Department of Pediatrics, Division of Pediatric Nephrology, Istanbul University Faculty of Medicine, Istanbul, Turkey

## Abstract

**Bakcground:**

The aim of this study was to evaluate the efficacy and safety of citrate versus heparin anticoagulation for CRRT in critically-ill children.

**Methods:**

This retrospective comparative cohort reviewed the clinical records of critically-ill children undergoing CRRT with either RCA or systemic heparin anticoagulation. The primary outcome measure was hemofilter survival time. Secondary outcomes included the comparison of complications and metabolic disorders.

**Results:**

A total of 131 patients (55 RCA and 76 systemic heparin) were included, in which a cumulative number of 280 hemofilters were used (115 in RCA with 5762 h total CRRT time, and 165 in systemic heparin with 6230 h total CRRT time). Hemofilter survival was significantly longer for RCA (51.0 h; IQR: 24–67 h) compared to systemic heparin (29.5 h; IQR, 17–48 h) (*p* = 0.002). Clotting-related hemofilter failure occurred in 9.6% of the RCA group compared to 19.6% in the systemic heparin group (*p* = 0.038). Citrate accumulation occurred in 4 (3.5%) of 115 RCA sessions. Hypocalcemia and metabolic alkalosis episodes were significantly more frequent in RCA recipients (35.7% vs 15.2%, *p* < 0.0001; 33.0% vs 19.4%, *p* = 0.009).

**Conclusion:**

RCA is a safe and effective anticoagulation method for CRRT in critically-ill children and it prolongs hemofilter survival.

**Impact:**

- RCA is superior to systemic heparin for the prolongation of circuit survival (overall and for clotting-related loss) during CRRT.
- These data indicate that RCA can be used to maximize the effective delivery of CRRT in critically-ill patients admitted to the PICU.
- There are potential cost-saving implications from our results owing to benefits such as less circuit downtime and fewer circuit changes.

## Introduction

Continuous renal replacement therapy (CRRT) is the most commonly used renal replacement therapy (RRT) in critically-ill children. It is used in various indications, including acute kidney injury (AKI), severe electrolyte imbalance/metabolic acidosis, refractory or diuretic-resistant fluid overload, intoxications, and inborn metabolic disorders.^1,2^ Therapeutic efficacy is associated with a number of factors, one of which is circuit survival (CS) that depends on anticoagulation.^3^ During CRRT, ideal anticoagulation should be able to sustain the circuit while causing minimal side effects on circulation. It should be safe, available, consistent, easily monitored, and reversible. Premature clotting of the CRRT circuit can lead to increased workload and health care costs, as well as adverse outcomes for the patient.^4,5^ Thus, efficient anticoagulation protocols are required to prevent clotting and prolong circuit life (CL).

In clinical practice, systemic heparin anticoagulation and regional citrate anticoagulation (RCA) are the two main anticoagulation strategies for CRRT. The major advantages of using heparin are its low cost, ease of administration, monitoring, and reversibility. However, the increased risks for bleeding with heparin use is a major concern in critically-ill children who are predisposed to bleeding for many reasons such as surgical procedures, trauma, severe liver dysfunction, and thrombocytopenia.^6^ RCA is favoured by many intensivist as it avoids systemic anticoagulation and inhibits the clotting cascade by chelating ionized calcium (iCa) preventing the initiation of the coagulation cascade also thrombin formation.^7^

RCA was studied for the first time by Mehta et al who examined a group of 18 adults with acute renal failure. The study revealed the superiority of RCA over heparin in terms of bleeding risk and thrombocytopenia.^8^ In subsequent adult studies, RCA has been demonstrated to be superior to systemic heparin anticoagulation based on reduced clotting, prolonged hemofilter life, and lower risk of bleeding.^7–13^ Indeed, the 2012 kidney disease improving global outcomes (KDIGO) guidelines recommended using RCA for adult patients at increased risk of bleeding –given that there are no contraindications for citrate infusion.^10^ In critically-ill children requiring dialysis, citrate anticoagulation was used successfully by Bunchman et al. in the early 2000s.^14^ However, when compared to adults, research concerning critical-ill children receiving CRRT is limited in terms of evaluating RCA for hemofilter survival, treatment efficacy, and safety, particularly in Turkey. Furthermore, available studies are mostly comprised of limited patient series and some results are conflicting.^7,9,15–22^ This study aims to compare RCA and systemic heparin in pediatric CRRT to assess safety and efficacy by examining hemofilter survival, complications, and metabolic disturbance.

## Materials and methods

### Study design

This study was conducted in a multidisciplinary tertiary PICU of Prof. Dr. Suleyman Yalcin City Hospital in Istanbul, Turkey. Our research design is based on a retrospective single-center cohort. The study was approved by Medical Ethics Committee of our hospital (Registration number: 2021/0017). Patient consent was waived due to retrospective nature of the study.

### Study population

All critically-ill children who were admitted to our general PICU from April 2015 to January 2021 and received CRRT with either systemic anticoagulation (heparin) or RCA were assessed for enrolment. Patients were excluded if they fulfilled at least one of the following criteria: (i) insufficient data, (ii) received anticoagulation therapy for therapeutic purposes during CRRT or up to 24 h (h) before the start of CRRT, (iii) received an RRT modality other than CRRT (intermittent or peritoneal hemodialysis), (iv) underwent RRT for various other reasons, such as chronic renal failure, before admission to the unit.

**Fig. 1:**
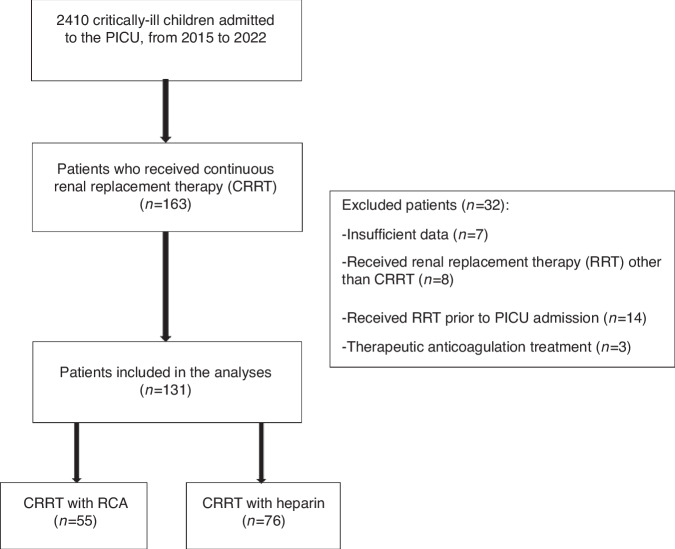
Flowchart of the study. PICU Pediatric intensive care unit, CRRT Continuous renal replacement therapy, RRT Renal replacement therapy, RCA Regional citrate anticoagulation.

The subjects were divided into two groups: one group comprising patients who received RCA and the second group comprising of those who received systemic anticoagulant with heparin (Fig. 1).

### Data collection and definitions

CRRT was performed according to locally-defined protocols. Demographic data, laboratory results, and CRRT-specific data were extracted from medical records or any other pertinent clinical data storage (physical files, notes etc.). Age, weight, sex, presence of comorbidities, diagnosis, Pediatric Risk of Mortality (PRISM) III score, and risk factors associated with bleeding were recorded at admission to the PICU. Admission diagnoses were classified into eleven groups as follows: renal disease, hemato-oncologic disease, sepsis, pneumonia, respiratory failure, liver failure, cardiac arrest, metabolic disease, cardiopathies, intoxication, acute abdomen, and others. Identified risk factors for bleeding were admission due to major surgery or trauma, thrombocytopenia/coagulopathy, hematologic malignancies, sepsis, respiratory failure (requiring invasive mechanical ventilation), hepatic failure, extracorporeal membrane oxygenation (ECMO), and higher PRISM III score at admission.^23,24^

Clinical data collected at CRRT initiation were as follows: indication for CRRT initiation, percentage of fluid overload (%FO), urine output (UO), need for invasive mechanical ventilation, presence of multiple organ dysfunction syndrome (MODS), need for ECMO, vasoactive inotropic support / vasoactive inotrope score (VIS), and stage of AKI. The indications for CRRT initiation were classified as electrolyte or acid-base imbalance, AKI, FO, hyperammonemia, acute exacerbation of inborn metabolic disorders, intoxication, tumor lysis syndrome, and rhabdomyolysis. The indications for commencing CRRT were identified from the documentation of the attending intensivist. Survival was defined as surviving until pediatric intensive care unit (PICU) discharge.

%FO at CRRT initiation was calculated using the following formula: (total fluid intake [L] – total fluid output [L] / (PICU admission weight [kg]) × 100.^25^ Analysis was performed separately for three FO categories (<10%, 10–20%, and >20%), which were defined based on previous studies.^26,27^ UO was calculated for the 24 h before the initiation of CRRT. VIS was calculated using the following formula: (dose of dopamine + dobutamine + [100 × epinephrine] + [100 × norepinephrine] + [10 × milrinone] [all in microgram/kg/min] + [10.000 × vasopressin] [U/kg/hr]).^28^ AKI was classified using KDIGO criteria based on changes in serum creatinine. If baseline serum creatinine was not available, we instead utilized the value of UO in the last 24 h before CRRT initiation.^10^ Finally, MODS was defined as the presence of at least three failed organs, according to the guidelines put forth by the Pediatric Sepsis Consensus Conference.^28^

Biochemical variables collected at the initiation of CRRT were as follows: creatinine, urea, hemoglobin, platelet count, pH/arterial blood gases, lactate, serum electrolytes, prothrombin time (PT) (sec), activated partial thromboplastin time (aPTT) (sec) and international normalization ratio (INR).

The CRRT operating parameters were as follows: total CRRT time, the number of hemofilters used and survival, the size of hemofilters and catheters, CS time (irrespective of cause), CRRT parameters (pump flow rate, dialysate/replacement flow rates), and the calcium infusion rate and citrate dosage in RCA recipients. Reasons for disconnection/hemofilter failure were categorized as (i) clotting in the circuit, (ii) vascular access malfunction, (iii) scheduled replacement after 72 h, (iv) end of CRRT treatment, and (v) technical issues/alarms.

CS time was defined as the time from the beginning of CRRT with the corresponding filter until filter replacement (until the upper limit for regular filter replacement; 72 h) or termination of CRRT session due to various reasons including achievement of treatment goals or death or clotting & non-clotting events (vascular access malfunction, technical reasons).

Electrolyte-related and metabolic disturbances during CRRT were recorded and categorized as follows: hypocalcemia (iCa^++^ < 0.9 mmol/L), hypercalcemia (iCa^++^, > 1.25 mmol/L), hyponatremia (Na < 130 mmol/L), hypernatremia (Na > 145 mmol/L), hypomagnesemia (< 1.5 mg/dl), hypophosphatemia (< 2.5 mg/dL), metabolic acidosis (pH < 7.36 or base excess [BE] < −3), and metabolic alkalosis (pH > 7.46 or BE > + 3). Citrate accumulation (CA) was defined as a ratio of total calcium (totCal^++^)-to-ionized calcium (iCal + +) of > 2.5 for longer than 48 h with high anion gap metabolic acidosis.^29^

The volume of packed red cells transfused during CRRT (and after circuit clotting) was recorded for each circuit. Transfusions were initiated in response to hemoglobin concentration measurements obtained from regular blood sampling, with a threshold of 7.0 g/dL during CRRT.^30^ If the reason for hemoglobin decrease below 7.0 g/dL was circuit clotting, the indication for transfusion was defined to be due to the circuit clotting. Bleeding site (catheter insertion site, gastrointestinal site etc.) and bleeding severity were recorded. Severe bleeding was defined as bleeding associated with hypotension or tachycardia, bleeding that necessitated red blood cell transfusion, or a 2 g/dl drop in hemoglobin within 24 h.

A number of other variables that may affect circuit survival, as suggested by prior research,^5,31,32^ were also assessed. These variables included use of femoral vein catheter, low patient weight (< 10 kg), small hemofilter size (60 m^2^ or 93 m^2^), lower pump flow rate (< 100 mL/min) and use of systemic anticoagulation with heparin.

### CRRT protocol and anticoagulation method

Initial CRRT was performed according to our institutional protocol. All CRRT treatments were performed using a Prismaflex HF20, M60 and M100 control unit (Gambro, Sweden and Baxter). Continuous veno-venous hemodiafiltration was administered to all subjects. Venous access was obtained by dual lumen hemodialysis catheters (7 F to 12 F) depending on the age and weight of the child.

During treatments, blood flow rate was set 3–8 ml/kg/min with respect to patient weight, catheter size and filter surface. Total clearance rate was set between 2–3 L/1.73 m^2^/hr for the majority of patients, while higher total clearance rates (up to a maximum of 5 L/1.73 m^2^/hr) were reached in cases with specific indications, such as intoxication or hyperammonemia. The dialysate flow rate, replacement fluid rate and ultrafiltration rate values were customized based on diagnosis, hemodynamic parameters and FO values. In both anticoagulation strategies, while regulating dialysate and replacement flows, care was taken not to increase the filtration fraction above 25%.

Poly aryl ethylene sulphone (PAES) membranes (Prismaflex HF20; circuit volume 60 ml, surface area 0.2 m^2^) or AN69 membranes (Prismaflex M60; circuit volume 93 ml, surface area 0.6 m^2^ and Prismaflex M100; circuit volume 152 ml, surface area 0.9 m^2^) were used. Children with a body weight of < 10 kg underwent CRRT using a Prismaflex HF20 set, those with a body weight between 10 kg and 25 kg received the Prismaflex M60 set, while those exceeding 25 kg received a Prismaflex M100 set.

Heparinized saline (5 U/mL) was used for hemofilter priming. In patients with high risk for hemorrhage, the circuit was primed with physiological saline only. An overall exception was use of blood to prime the circuit in children with a bodyweight of less than 10 kg and those with hemoglobin values below 10 g/dL. For this purpose, erythrocyte suspension was diluted 1-to-1 with saline.

### Anticoagulation method

Before March 2017, RCA was not performed at our center; thus, the anticoagulation protocol was systemic heparin in all patients admitted before this date. After this date, anticoagulation was chosen based on age, clinical characteristics and diagnosis.

For heparin, infusion was started with 10 international units (IU)/kg/hr pre-filter. The heparin dose was subsequently adjusted toward a target postfilter activated clotting time (ACT) of 180–220 s. Dialysis and replacement solutions were the Dialisan (DVVHD BG2D, Baxter) and Prism0calB22 (Baxter) solutions.

For citrate administration, a Prismaflex system (Baxter), employing an automated RCA method with commercially available citrate-buffered solution (Prismocitrate 18/0, Baxter) and compatible bicarbonate dialysate solutions (Prism0cal B22, Baxter), was used. Citrate flow was coupled to blood flow and adjusted by the CRRT device to achieve the prescribed citrate dose (3 mmol per L of blood). CRRT was performed with pre-filter citrate anticoagulation and post-filter replacement fluid. Citrate effect was neutralized using a continuous calcium infusion, calcium gluconate 10% 50/50 with dextrose 5% (116 mmol/L). Calcium infusion was administered through a different central venous line. Only when a separate central line was not available, we administered calcium infusion through the return line of the circuit. An initial infusion rate of 1 mL/kg/h was used to maintain ionized calcium blood level. Calcium compensation was determined according to systemic ionized calcium values. The filter target iCa^++^ level was between 0.25 and 0.35 mmol/L and the patient’s iCa^++^ target was between 1 and 1.2 mmol/L. The monitoring of iCa^++^ concentrations was performed at several time points to ensure maintenance of target concentrations: at the 30th minute, the first, second and fourth hour of therapy, and then routinely every 4 h. Patient calcium levels were also checked one hour after any change in blood flow, citrate concentration, or dialysis. Biochemical indicators and electrolytes were checked every 12–24 h in both anticoagulation strategies.

In the presence of CA, interventions were made to reduce blood flow rate or citrate flow rate/dose, or to increase dialysate flow rate to facilitate citrate clearance. In order to correct ionized hypocalcemia, calcium infusion rate was increased. In case of metabolic alkalosis, in addition to the interventions performed for CA, we administered 0.9% sodium chloride infusion as pre- or post-replacement fluid.^29,33^

### Statistical analysis

The demographic and clinical characteristics of the study subjects were described as well as variables associated with the filter. Characteristics of patients, as n (percent) or median (interquartile range [IQR]) for categorical and continuous variables, respectively, and were compared among groups using chi-square or Mann-Whitney tests, as appropriate. Results were evaluated with a significance value of *p* < 0.05. Kaplan-Meier survival analysis was applied for survival curves. The median survival for each group, with its respective 95% confidence interval (CI), was reported, and a comparison of the curves was tested using a log-rank test. The magnitude of the effect of the anticoagulant on clotting in the filter was analyzed using Cox’s non-parametric proportional hazards model, which was adjusted for potential confounding variables (size of the filter and pump flow) that could affect the lifetime of the filter. The statistical analysis was performed using the SPSS (version 23.0) software (SPSS Inc., Chicago, IL).

## Results

During the 6-year period, 2410 patients were admitted to the PICU and 163 patients received CRRT treatment. All 163 patients were analyzed in the study. Thirty-two children were excluded based on mentioned criteria and a final total of 131 children were included in final analysis. The flow-chart describing participant inclusion is shown in Fig. 1.

### Description of the study population

A total of 131 patients (55 in the RCA group and 76 in the systemic heparin group) were included in the analysis. The demographic and clinical characteristics of the patients were largely similar in the two groups (Table 1), except for the presence of liver failure and hyperammonemia, which were more frequent among heparin recipients (*p* = 0.010, *p* = 0.044), and the frequency of tumor lysis syndrome which was more common in the RCA group (*p* = 0.029). There was no significant difference in length of PICU stay and PICU survival.

**Table 1:** Patients’ variables and differences between sitrat versus heparin.

Variable	All Patients (*n* = 131)	Citrate group (*n* = 55)	Heparin group (*n* = 76)	*p*
Age (yr), median (IQR)	3.9 (1.3–11.0)	5.2 (1.3–11.3)	3.0 (1.3–7.3)	0.089
Male sex, *n* (%)	65 (49.6)	28 (50.9)	37 (48.7)	0.941
Weight (kg), median (IQR)	16 (11–32)	21 (11–42)	13.3 (10.8–23)	0.051
Weight < 10 kg, *n* (%)	24 (18.3)	9 (16.4)	15 (19.7)	0.792
PRISM III score, median (IQR)	15 (9–24)	14 (10–24)	17 (9–24)	0.976
Comorbidity, *n* (%)	68 (51.9)	30 (54.5)	38 (50.0)	0.736
Risk factor for bleeding, *n* (%)	53 (40.5)	25 (45.5)	28 (36.8)	0.417
Diagnosis at admission to PICU, *n* (%)				
Renal disease	43 (32.8)	16 (29.1)	27 (35.5)	0.093
Hemato-oncologic disease	19 (14.5)	9 (16.4)	10 (13.2)	0.819
Sepsis	14 (10.7)	7 (12.7)	7 (9.2)	0.744
Pneumonia / Respiratory failure	12 (9.2)	6 (10.9)	6 (7.9)	0.650
Liver failure	9 (6.9)	0 (0.0)	9 (11.8)	0.010
Cardiac arrest	9 (6.9)	7 (12.7)	2 (2.6)	0.096
Metabolic disease	8 (6.1)	2 (3.6)	6 (7.9)	0.157
Cardiopathies	4 (3.1)	2 (3.6)	2 (2.6)	1.000
Intoxication	6 (4.6)	3 (5.5)	3 (3.9)	1.000
Acute abdomen	2 (1.5)	0 (0.0)	2 (2.6)	0.509
Other	5 (3.8)	3 (5.5)	2 (2.6)	0.655
CRRT indication				
Electrolyte / acid base disturbance	48 (36.6)	19 (34.5)	29 (38.2)	0.810
Acute kidney injury	42 (32.1)	21 (38.2)	21 (27.6)	0.277
Fluid overload	20 (15.3)	8 (14.5)	12 (15.8)	1.000
Hyperammoniemia	10 (7.6)	1 (1.8)	9 (11.8)	0.044
Acute attack of metabolic disease	6 (4.6)	1 (1.8)	5 (6.6)	0.400
Tumor lysis syndrome	4 (3.1)	4 (7.3)	0 (0.0)	0.029
Rhabdomyolysis	1 (0.8)	1 (1.8)	0 (0.0)	0.420
Clinical variables at initiation of CRRT				
% Fluid overload, median (IQR)	6.7 (0.0–18.8)	11.5 (0.0–18.8)	4.0 (0.0–17.95)	0.296
% Fluid overload, *n* (%)				
< 10%	73 (55.7)	25 (45.5)	48 (63.2)	0.066
10–20%	29 (22.1)	16 (29.1)	13 (17.1)	0.156
> 20%	29 (22.1)	14 (25.5)	15 (19.7)	0.572
Urine output (mL/kg/hr), median (IQR)	0.3 (0.0–0.4)	0.2 (0.0–0.4)	0.3 (0.0–0.5)	0.281
Mechanical ventilation, *n* (%)	80 (61.1)	33 (60.0)	47 (61.8)	0.975
Mechanical ventilation (day), median (IQR)	8 (2–15)	8 (3–24)	7 (2–12)	0.486
Multiple organ dysfunction syndrome, *n* (%)	59 (45.0)	21 (38.2)	38 (50.0)	0.244
Vasoactive support, *n* (%)	71 (54.2)	30 (54.5)	41 (53.9)	1.000
Vasoactive inotrope score, median (IQR)	30 (20–40)	31.3 (22.5–45)	25 (15–35)	0.153
Acute kidney injury stage 2 and 3, *n* (%)	66 (50.4)	29 (52.7)	37 (48.7)	0.780
Isolated acute kidney injury, *n* (%)	22 (16.8)	7 (12.7)	15 (19.7)	0.411
Insertion site of access catheters				
Internal jugular	59 (45.0)	29 (52.7)	30 (39.5)	0.185
Subclavian	15 (11.5)	7 (12.7)	8 (10.5)	0.910
Femoral	57 (43.5)	19 (34.5)	38 (50.0)	0.114
Laboratory variables at initiation of CRRT, median (IQR)				
Creatinine (mg/dL)	2.08 (0.75–3.8)	2.1 (0.9–3.58)	1.62 (0.63–3.94)	0.218
Urea (mg/dL)	83 (42–150)	101 (47–146)	76 (38–150)	0.508
Hemoglobin (gr/dL)	8.8 (7.3–10.7)	8.9 (7.2–10.7)	8.8 (7.4–10.7)	0.913
Platelet count, (X 10^3^/µL)	103 (45.6–226)	69 (32–195)	113.5 (57–231.5)	0.074
pH	7.2 (7.09–7.3)	7.17 (7.08–7.3)	7.2 (7.1–7.3)	0.567
Lactate	3.5 (1.5–6.0)	4.0 (1.6–9.0)	3.5 (1.4–5.6)	0.121
Potassium, (mmol/L)	4.2 (3.8–4.8)	4.3 (3.8–5.0)	4.0 (3.8–4.6)	0.127
Sodium, (mmol/L)	138 (134–141)	137 (133–141)	138 (134–140)	0.859
Phosphor, (mg/dl)	4.6 (3.4–6.2)	4.8 (3.4–6.3)	4.55 (3.3–6.15)	0.346
Magnesium, (mg/dl)	2.05 (1.77–2.33)	2.0 (1.75–2.33)	2.05 (1.78–2.37)	0.836
Total calcium, (mg/dL)	8.2 (7.6–8.9)	8.0 (7.4–8.6)	8.3 (7.65–9.0)	0.125
Ionized calcium, (mmol/L)	1.03 (0.98–1.09)	1.03 (0.98–1.1)	1.03 (0.98–1.08)	0.445
Prothrombin time (sec)	17.3 (15.5–21.0)	17.1 (15.2–22.0)	17.8 (15.5–20.7)	0.987
International normalization ratio	1.38 (1.19–1.69)	1.37 (1.18–1.69)	1.38 (1.2–1.69)	0.789
Activated partial thromboplastin time (sec)	32.6 (30.3–39.5)	32.0 (30.0–39.4)	33.9 (30.4–40.1)	0.663
Length of PICU stay (days), median (IQR)	7 (3–13)	8 (4–14)	6 (3–12)	0.226
PICU mortality, *n* (%)	37 (28.2)	19 (34.5)	18 (23.7)	0.244

*PRISM* Pediatric risk of mortality score, *PICU* Pediatric intensive care unit, *CRRT* Continuous renal replacement therapy, *IQR* Interquartile range.

### CRRT and hemofilter characteristics

A total of 11,992 h of dialysis therapy (RCA group 5762 h, heparin group 6230 h) were included in the analysis (Table 2). Cumulatively, a total of 280 hemofilters/circuits were used in all therapies, 115 in the RCA group and 165 in the systemic heparin group.

**Table 2:** Characteristics of CRRT in children treated with citrate and heparin.

Variables	All Patients (*n* = 131)	Citrate group (*n* = 55)	Heparin group (*n* = 76)	*p*
Total CRRT time (hr)	11992	5762	6230	
CRRT time (hr) per patient, median (IQR)	64 (32–115)	70 (40–144)	56 (30–96)	0.187
Total number of hemofilters	280	115	165	
Number of filters per patient, median (IQR)	2 (1–3)	2 (1–3)	2 (2–2)	1.000
Median circuit lifetime (hr), median (IQR)	40 (20–57)	51 (24–67)	29.5 (17–48)	0.024
Median circuit lifetime for clotting hemofilter (h)	19 (12–32)	38 (24–50)	15 (10–23)	0.001
Filter size (m^2^)				
60	45 (34.4)	15 (27.3)	30 (39.5)	0.206
93	46 (35.1)	18 (32.7)	28 (36.8)	0.763
152	40 (30.5)	22 (40.0)	18 (23.7)	0.070
Pump flow rate, (mL/min), median (IQR)				
< 100	87 (66.4)	32 (58.2)	55 (72.4)	0.131
≥ 100	44 (33.6)	23 (41.8)	21 (27.6)	
Dialysate flow rate, (mL/min), median (IQR)	600 (400–900)	800 (500–900)	600 (400–800)	0.084
Dialysate flow rate per kg, (mL/kg), median (IQR)	36.3 (26.6–45.4)	33.4 (23.5–42.8)	39.4 (29.3–47.1)	0.071
Replacement flow rate, (mL/min), median (IQR)	500 (300–750)	500 (300–800)	450 (300–600)	0.211
Replacement flow rate per kg, (mL/min), median (IQR)	25.0 (20.0–33.3)	24.0 (19.1–29.6)	27.7 (22.5–34.8)	0.006
Calcium infusion flow rate (mmol/h)	3.08 (1.32–5.78)	3.08 (1.32–5.78)	NA	NA
Calcium infusion flow rate per kg (mmol/kg/h)	0.85 (0.08–0.10)	0.85 (0.08–0.10)	NA	NA
Citrate dose (mmol/L), median (IQR)	4.0 (3.5–4.0)	4.0 (3.5–4.0)	NA	NA
T/I calcium ratio, median (IQR)	2.04 (1.88–2.20)	2.04 (1.88–2.20)	NA	NA
Dialysis catheter size (French)				
7	17 (13.0)	5 (9.1)	12 (15.8)	0.388
8	42 (32.1)	16 (29.1)	26 (34.2)	0.667
9	24 (18.3)	9 (16.4)	15 (19.7)	0.792
10	20 (15.3)	10 (18.2)	10 (13.2)	0.587
11	21 (16.0)	12 (21.8)	9 (11.8)	0.195
12	7 (5.3)	3 (5.5)	4 (5.3)	1.000

*CRRT* Continuous renal replacement therapy, *IQR* Interquartile range, *T* Total calcium, *I* Ionized calcium.

**Fig. 2:**
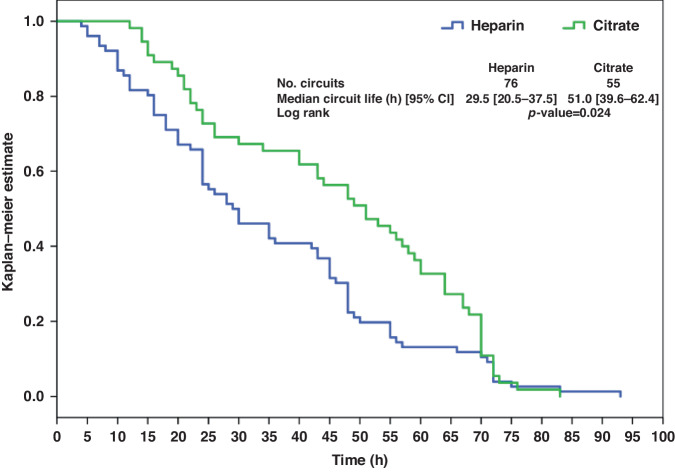
Kaplan–Meier survival curve for overall circuit lifetime with anticoagulation using citrate and heparin. The figure illustrates the Kaplan–Meier survival curve depicting the overall circuit lifetime in patients undergoing anticoagulation with citrate and heparin. Each line represents a different anticoagulation strategy, with symbols indicating censoring events.

**Fig. 3:**
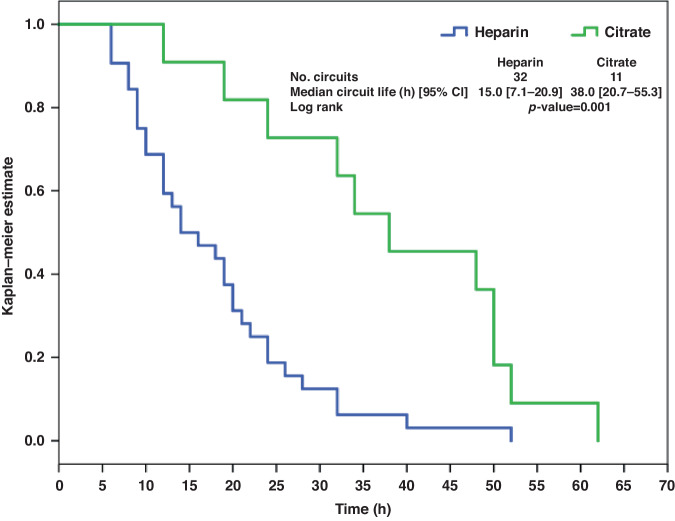
Kaplan–Meier survival curve for overall circuit lifetime in subgroup analysis of clotted filters. This figure presents the Kaplan–Meier survival curve for overall circuit lifetime in the subgroup analysis including only clotted filters. Each line represents a different subgroup, with symbols indicating censoring events.

Kaplan Meier analysis showing overall CS time (for all reasons of circuit disconnection) was significantly higher in the RCA group compared to the systemic heparin group [51.0 h (95% CI 39.6–62.4 h) vs. 29.5 h (95% CI 20.5–37.5 h); *p* = 0.024] (Fig. 2). Similarly, CS time limited by clotting hemofilter was significantly higher in the RCA group [38 h (95% CI 20.7–55.3 h) vs. 15 h (95% CI 7.1–20.9 h); *p* = 0.001] (Fig. 3).

In the RCA group, there were 11 events of hemofilter removal due to clotting. The systemic heparin group presented with 32 such events, which was significantly higher than the RCA group (respectively; 19.4% vs. 9.6%, *p* = 0.03). Other reasons of circuit disconnection were not different between groups (Table 3).

**Table 3:** Reasons for hemofilter disconnection/failure in the heparin and citrate group.

Causes, n (%)	All Hemofilter (*n* = 280)	Citrate hemofilter (*n* = 115)	Heparin hemofilter (*n* = 165)	*p*
Clotting	43 (15.4)	11 (9.6)	32 (19.4)	0.038
Vascular access malfunction	32 (11.4)	12 (10.4)	20 (12.1)	0.806
^a^Scheduled filter replacement after 72 h	77 (27.5)	38 (33.0)	39 (23.6)	0.083
End of CRRT treatment	122 (43.6)	51 (44.3)	71 (43.0)	0.827
^b^Technical issues/alarms	6 (2.1)	3 (2.6)	3 (1.8)	0.976

*CRRT* Continuous renal replacement therapy.

^a^Adviced maximum duration of hemofilter use according to manufacturer.

^b^Incorrect scale balanced causes blood flow stop or anaphylactoid reaction to the hemofilter.

Cox’s proportional risk analysis showed that the risk of hemofilter clotting (median CS time limited by clotting hemofilter) was 3.3-fold higher with the use of systemic heparin compared to RCA (HR: 3.30; 95% CI: 1.57–7.09; *p* = 0.002). Dialysis catheter placement, low weight, small filter size and low pump flow rates were not significantly associated with hemofilter clotting (Table 4).

**Table 4:** Cox’s proportional risk analysis of risk of hemofilter clotting.

Half-life of hemofilters				
Variables	Hazard ratio	Standard error	*p*-value	95% Confidence Interval
Femoral vein catheter	1.627	0.344	0.158	0.828–3.195
Weight < 10 kg	1.062	0.600	0.920	0.328–3.444
Filter size 60 m^2^	5.017	0.919	0.079	0.829–30.380
Filter size 93 m^2^	5.045	0.822	0.059	1.007–25.268
Pump flow rate < 100	0.346	0.838	0.205	0.067–1.787
Heparin anticoagulation	3.300	0.385	0.002	1.570–7.098

### Safety issues

#### Bleeding complication

Among all subjects, 20 episodes of severe bleeding (6 in the RCA group and 14 in the systemic heparin group) occurred. The frequency of severe bleeding was similar in the two groups (*p* = 0.35). There was no significant difference in red cell transfusion ratio. The RCA group had a significantly lower frequency of circuit clotting-related transfusions-to-total transfusions compared to the heparin group (12.5% vs. 42.5%, respectively) (*p* < 0.001) (Table 5).

**Table 5:** Red cell transfusion rates and bleeding complication.

Variables	All patient (*n* = 131)	Citrate group (*n* = 55)	Heparin group (*n* = 76)	*p*
Red blood cell transfused patient, *n* (%)	87 (66.4)	32 (58.2)	55 (72.4)	0.131
Transfused red blood cell units during CRRT, median (IQR)	1.0 (0.8–2.0)	1.0 (0.8–2.0)	1.0 (0.7–1.5)	1.000
Number of transfusion after circuit clotting **/** total number of transfusions, *n* (%)	39/137 (28.5)	8/64 (12.5)	31/73 (42.5)	< 0.0001
Severe bleeding event, *n* (%)	20 (15.3)	6 (10.9)	14 (18.4)	0.350
Type of bleeding event, *n* (%)				
Catheter insertion	17 (13.0)	4 (7.3)	13 (17.1)	0.165
Gastrointestinal bleeding	7 (5.3)	2 (3.6)	5 (6.6)	0.698

*CRRT* Continuous renal replacement therapy, *IQR* Interquartile range.

#### Metabolic complications

The most frequent complications were hypocalcemia (35.7% in the RCA group, 15.2% in the heparin group) and metabolic alkalosis (33% in the RCA group, 19.4% in the heparin group). Both of these complications were significantly more common with RCA than systemic heparin (*p* < 0.001 and *p* = 0.009). There were 4 cases of CA (3.5%). Median totCa^++^ to iCal^++^ index (CaI) was 2.24 (IQR: 1.92- 2.24), and the maximum CaI was 2.59 with metabolic acidosis. The groups were similar in terms of other electrolyte imbalances (hypercalcemia, hyponatremia, hypernatremia, hypomagnesemia, hypophosphatemia) (Table 6). No association was found between metabolic complications and citrate dosage, patient weight, dialysate, or replacement flow rate (*p* > 0.05, data not shown).

**Table 6:** Metabolic and electrolyte disturbances in study groups.

Variables, *n* (%)	Citrate group % (*n* = 115)	Heparin group % (*n* = 165)	*p*
Hypocalcemia	**41 (35.7)**	**25 (15.2)**	< 0.0001
Hypercalcemia	3 (2.6)	0 (0.0)	0.068
Hyponatremia	0 (0.0)	0 (0.0)	NA
Hypernatremia	18 (15.7)	16 (9.7)	0.189
Hypomagnesemia	13 (11.3)	13 (7.9)	0.446
Hypophosphatemia	35 (30.4)	42 (25.5)	0.359
Metabolic alkalosis	38 (33.0)	32 (19.4)	0.009
Citrate accumulation	4 (3.5)	NA	NA

## Discussion

The key findings of this study are that RCA is superior to systemic heparin for the prolongation of CS (overall and for clotting-related loss) during CRRT. RCA also reduces hemofilter failure due to clotting and need for transfusion after circuit clotting. It has manageable and limited side effects like hypocalcemia, CA and metabolic alkalosis. Therefore, the current study confirms that RCA is relatively safe and can prolong the survival of hemofilters as compared to heparin anticoagulation, which conforms with the data provided by several pediatric studies. Our data specifically indicate that RCA can be used to maximize the effective delivery of CRRT in critically-ill patients admitted to the PICU. There are also potential cost-saving implications from our results owing to benefits such as less circuit downtime and fewer circuit changes.

Although no significant difference was found between RCA and systemic heparin in some adult studies evaluating anticoagulation efficacy, RCA has been emphasized as a more effective method in recent years, and this has resulted in the recognition of RCA as the recommended approach.^6,11,12,34,35^ In the meta-analysis of adult studies conducted by Liu et al, it was stated that the use of RCA prolonged filter life by an average of 15.6 h compared to systemic heparin.^11^ Recently, in a randomized controlled study conducted by Zarbock et al in adults (596 patients), it was reported that RCA prolonged filter life (47 h vs 26 h) compared to systemic heparin.^35^ Few studies have been carried out in children regarding the anticoagulation strategy in CRRT and most of these studies were conducted in small series. Nonetheless, in the majority of these studies, RCA has been shown to prolong mesh life compared to systemic heparin.^17–19,22,31,36–40^ In a prospective study by Brophy et al on 138 patients and 442 CRRT circuits, no significant difference was found between RCA and systemic heparin use in terms of circuit life (42.1 ± 27.1 h vs. 44.7 ± 35.9 h), albeit systemic heparin was found to cause increased risks for severe bleeding.^41^ In recent years, with the development of reliable dialysis devices with very small extracorporeal volumes, CRRT treatment has been used more frequently in young infants and newborns, as well as in adult and pediatric cases.^37,42,43^ Although the number of cases is limited, RCA can be used safely in newborns receiving CRRT. There are reported publications in this age groups.^16,42–45^ In the Critical Care Nephrology Section of The European Society of Paediatric and Neonatal Intensive Care (ESPNIC), as a result of the evaluation of current pediatric and neonatal studies, it was reported that RCA may extend circuit life by reducing coagulation.^46^

In our study, median hemofilter survival was 51.0 h (IQR: 24–67), which was similar to some pediatric studies,^17,18,36,40^ but shorter than those reported by other pediatric studies.^14,22,38^ These differences between the filter lifetimes in the studies may be due to variations in filter size, catheter site, citrate concentration used, targeted ionized calcium level, and age groups.^22,47^

CS time is dependent on many factors including clotting and non-clotting events (vascular access malfunction and technical issues/alarms etc.).^19,22^ In our study, the rate of hemofilter failure due to clotting was significantly higher in the systemic heparin group (19.4% vs. 9.4%). In addition, in our subgroup analysis, the median CS time for clotting hemofilters was significantly higher in the RCA group than in the systemic heparin group (15 h vs. 28 h). Similar results were found in other pediatric studies.^19,22,36^ Moreover, the CS time is claimed to be influenced by many factors, such as the patient’s clinical condition, coagulation status, the position and patency of the vascular access and catheter size, the choice of anticoagulant, CRRT modality, and filtration fraction.^5,31,32,47^ In our study, Cox regression did not yield evidence to support the literature in a number of factors, including patient weight (< 10 kg), filter size, catheter location, pump flow rate and the half-life of the clotted hemofilter. We found that the risk of hemofilter clotting was 3.3-fold higher with systemic heparin compared to citrate. This result was in support of our previously mentioned data and we can conclude that citrate can improve CS by reducing the likelihood of clotting. Therefore we can conclude that RCA is an efficient method in critically in children under CRRT with respect to progress of treatment.

The potential advantage of RCA for CRRT is that it may reduce the occurrence of systemic adverse events. The reported bleeding incidences associated with systemic heparin use in CRRT ranges from 10% to 50%, with bleeding mortality rates as high as 15%.^12,35,48^ In addition to various adult studies and meta-analyses, pediatric studies have also shown that RCA use in CRRT reduces bleeding risk by more than half compared to systemic heparin use.^4,6,7,35,39,41^ However, the effect of RCA in reducing the need for transfusion compared to systemic heparin use is controversial.^35^ This may be explained by the direct shutdown of systemic heparin in case of bleeding complications. However, discontinuation or interruption of heparin anticoagulation may increase the risk of clotting, leading to interruption of CRRT treatment and treatment failure. As opposed to previously reported results, we found that severe bleeding episodes were less frequent among RCA recipients (18.4% vs. 10.9%), albeit without statistical significance. Moreover, the need for red blood cell transfusions was higher in systemic heparin recipients compared to RCA, but again significance was not achieved. However, the frequency of blood transfusion requirement after circuit clotting (relative to all-cause transfusions) was significantly higher (42.5% vs 12.55%) among those who received systemic heparinization compared to RCA. In the pediatric study by Rico et al, it was observed that the use of RCA did not achieve a significant decrease in the incidence of serious bleeding events, but all bleeding events in the heparin group were systemic (gastrointestinal, central nervous system, intraabdominal) bleeding, whereas in the RCA group, up to 30% were local events (high flow catheter insertion site).^38^ In addition to prolonging filter life, RCA use may reduce filter clotting-related blood transfusions. This property might be crucial for small infants and may contribute to the reduction of costs and blood transfusions.

Since the main site of citrate metabolism is the liver, patients with hepatic failure and neonatal and small infants with immature liver function are risky groups for CA.^20^ In our study, we did not apply RCA in patients with hepatic failure because of concerns about citrate metabolism. However, in recent years, there are studies showing that it can be used safely in pediatric cases with hepatic failure and neonatal cases, but with strict monitoring.^20,41,49^ In a series of 50 cases including neonatal patients by Cappoli et al. it was reported that clinical findings of CA were observed in only 6 patients, all of whom had a calcium index of > 2.5. It is interesting that none of these 6 patients had liver failure.^40^ Rodriguez et al. reported that 70% of pediatric patients with liver failure experienced at least one episode of CA, but RCA had to be interrupted in only two such cases.^20^ In some pediatric studies, CA was not observed in any of the patients.^19,22,36,39^ In our study, CA was observed in 4 (3.5%) of 115 CRRT sessions with RCA. In adult studies, CA episodes have been reported at much lower rates (0%–0.7%) compared to pediatric studies.^6,35^ Since routine measurement of blood citrate level is not possible in practice, it should be kept in mind that CA can be estimated indirectly by totCa^++^ / iCa^++^ ratio ( > 2.5 mmol/L). In the present study, the definition of CA was based on an elevated totCa^++^ / iCal^++^ ratio accompanied by metabolic acidosis with high anion gap. However, in some studies, the totCa^++^ to iCal^++^ ratio of > 2.5 was accepted to establish CA diagnosis. However, the direct relationship between high totCa^++^ / iCa^++^ ratio and metabolic complications is controversial.^38^

The increased risk of CA in children undergoing RCA compared to adult patients may be explained by two factors. First, citrate infusions are often administered at much higher rates as compared to adults (on a mg per kg basis). This is because citrate infusion rates are based on blood flow and not patient weight. As discussed earlier, blood flow rates, especially in small children, are disproportionately high compared to adults when calculated in mL/min/kg, and therefore, citrate infusion rates must be increased to accommodate this difference.^7,50^ Second, lower citrate clearance rates have been reported in CRRT, compared to intermittent hemodialysis, and the rates are even lower when this therapy is used in children. In adults, clearance rates of 35–50% have been reported, compared with rates of only 20% in pediatric patients.^18^ In infants, strategies such as using lower concentrations of citrate (< 2.5 mmol/L) and targeting higher filter ionized calcium levels (< 0.5 mmol) and maintaining blood flow below a certain threshold (< 50 mL/min) can be used to reduce the risk of CA.^15,21,38^ In our study, none of the metabolic complications analyzed (including CA) were found to be associated with citrate dose or patient weight. We infer that the population could have other risk factors (metabolic capacity etc.) and/or triggers for complications apart from citrate use. All four episodes of CA were managed with conservative methods (such as decreasing blood flow rate, decreasing citrate flow rate, increasing dialysate rate) without altering the anticoagulation method.

The use of RCA is associated with metabolic complications, including possible life-threatening systemic hypocalcemia and metabolic alkalosis. Citrate is metabolized mainly in the liver into three moles of bicarbonate. Exposure to excessive citrate results in metabolic alkalosis in cases with sufficient liver metabolic capacity. This can be addressed by administering 0.9% sodium chloride infusion as pre- or post-replacement fluid, in addition to the conservative methods used for CA.^29,33,39^ Hypocalcemia, one of the most dangerous metabolic complications associated with RCA, occurs through two mechanisms. The first is the elevation of dialyzable calcium fraction due to the citrate-calcium complex. The second is the continuous binding of calcium by free citrate efflux, as the citrate clearance applied before the filter remains at the level of 35–50%.^51^

Frequency of these metabolic complications is variable and heterogeneous in both adult and pediatric studies.^6,18^ Similarly, the reported frequency of metabolic complications associated with the use of RCA is variable in both adult and pediatric population studies. In a meta-analysis, it was reported that hypocalcemia was 3.9 times more common with RCA, but no adverse events related to hypocalcemia were observed. Also, no significant difference was observed in the frequency of metabolic alkalosis.^11^ Zarbock et al. reported that RCA use did not pose a risk for the development of severe hypocalcemia (1.4% vs 0.3%), but increased the frequency of severe alkalosis (2.4% vs 0.3%) in adults.^35^ In pediatric studies, there is a wide range of reported frequencies for both metabolic alkalosis (2.0% to 86.5%) and hypocalcemia (0% to 60%) with RCA.^19,22,36,38,40,50^ In our study, hypocalcemia (35.7%) and metabolic alkalosis (33%) were significantly more common in RCA recipients compared to heparin recipients (15.2% vs. 19.4%, respectively).

These complications were diagnosed early by close monitoring of acid base status and calcium level, and were managed with conservative methods mentioned above. Thus, metabolic complications in patients treated with RCA should not be considered as a drawback, since they are mild and easily resolved. An important strategy to reduce metabolic complications associated with RCA is to reduce citrate exposure by lowering citrate infusion rate and dosage.^52–54^ Poh et al reported that applying a citrate protocol at a dose of 2.5 mmol/L reduced citrate-related metabolic complications without causing a decrease in anticoagulation effectiveness compared to 3 mmol/L.^54^

In the literature, the mortality rate in pediatric CRRT patients undergoing RCA varies between 16.7% and 50%, and in the majority of studies, no significant relationship has been found between anticoagulation choice and mortality.^9,39,40,49^ Overall mortality in our study was 28.2% and frequencies were similar in the two groups.

This study has several limitations. First, this is a single-center retrospective study. Second, we did not use RCA in patients with hepatic failure due to concerns regarding contraindication. Therefore, the systemic heparin and citrate groups are biased in this regard, potentially distorting findings. Third, patients from the 2015–2017 periods received standard anticoagulation with systemic heparin, because citrate was not used at our center before 2017. In March 2017, we started treating patients with RCA, and therefore, we cannot rule out a bias in this regard or an effect of increasing experience with CRRT. In addition, the sample size is smaller relative to adult studies which may subject the analyses to statistical bias; however, to our knowledge, this study has one of the largest sample sizes and longest CRRT duration (total hours) among pediatric CRRT studies comparing the efficacy and safety of these two anticoagulation methods.

Our data demonstrate that RCA could be more effective than systemic heparin for prolongation of CS during CRRT in critically-ill children. It appears to prolong CS with a lower incidence of clotting and blood transfusion after circuit clotting. We did not find any serious side effects of RCA, indicating that the efficacy is complemented by safety in pediatric CRRT. Notably, RCA may cause minimal metabolic and ionic imbalances which can be easily resolved. Further prospective studies are needed to assess the safety and efficacy of RCA in this population.

## Data Availability

Data included in this manuscript are available upon request by contacting with the corresponding author and will be freely available to any researcher wishing to use them for non-commercial purposes, without breaching participant confidentiality.

## References

[1] Sethi, S. K., Bunchman, T., Raina, R. & Kher, V. Unique considerations in renal replacement therapy in children: core curriculum 2014. *Am. J. Kidney Dis.* **63**, 329–345 (2014). 24161544 10.1053/j.ajkd.2013.08.018

[2] Garzotto, F., Zanella, M. & Ronco, C. The evolution of pediatric continuous renal replacement therapy. *Nephron. Clin. Pract.* **127**, 172–175 (2014). 25343844 10.1159/000363204

[3] Joannidis, M. & Oudemans-Van Straaten, H. M. Clinical review: Patency of the circuit in continuous renal replacement therapy. *Crit. Care.* **11**, 218 (2007). 17634148 10.1186/cc5937 PMC2206533

[4] Zhang, Z. & Hongying, N. Efficacy and safety of regional citrate anticoagulation in critically ill patients undergoing continuous renal replacement therapy. *Intensive Care Med.* **38**, 20–28 (2012). 22124775 10.1007/s00134-011-2438-3

[5] Del Castillo, J. et al. Circuit life span in critically ill children on continuous renal replacement treatment: a prospective observational evaluation study. *Crit. Care.* **12**, R93 (2008). 18657277 10.1186/cc6965 PMC2575577

[6] Wu, M. Y. et al. Regional citrate versus heparin anticoagulation for continuous renal replacement therapy: a meta-analysis of randomized controlled trials. *Am. J. Kidney Dis.* **59**, 810–818 (2012). 22226564 10.1053/j.ajkd.2011.11.030

[7] Davis, T. K., Neumayr, T., Geile, K., Doctor, A. & Hmeil, P. Citrate anticoagulation during continuous renal replacement therapy in pediatric critical care. *Pediatr. Crit. Care Med.* **15**, 471–485 (2014). 24777299 10.1097/PCC.0000000000000148

[8] Mehta, R. L., McDonald, B. R., Aguilar, M. M. & Ward, D. M. Regional citrate anticoagulation for continuous arteriovenous hemodialysis in critically ill patients. *Kidney Int.* **38**, 976–981 (1990). 2266683 10.1038/ki.1990.300

[9] Buccione, E. et al. Regional Citrate Anticoagulation and Systemic Anticoagulation during Pediatric Continuous Renal Replacement Therapy: A Systematic Literature Review. *J. Clin. Med.* **11**, 3121 (2022). 35683511 10.3390/jcm11113121 PMC9181744

[10] Group KDIGOGW. KDIGO clinical practice guideline for glomerulonephritis. *Kidney Int. Suppl.* **2**, 139–274 (2012).

[11] Liu, C., Mao, Z., Kang, H., Hu, J. & Zhou, F. Regional citrate versus heparin anticoagulation for continuous renal replacement therapy in critically ill patients: a meta-analysis with trial sequential analysis of randomized controlled trials. *Crit. Care.* **20**, 144 (2016). 27176622 10.1186/s13054-016-1299-0 PMC4866420

[12] Bai, M. et al. Citrate versus heparin anticoagulation for continuous renal replacement therapy: an updated meta-analysis of RCTs. *Intensive Care Med.* **41**, 2098–2110 (2015). 26482411 10.1007/s00134-015-4099-0

[13] Meersch, M. et al. Regional citrate versus systemic heparin anticoagulation for continuous renal replacement therapy in critically ill patients with acute kidney injury (RICH) trial: study protocol for a multicentre, randomised controlled trial. *BMJ Open* **9**, e024411 (2019). 30670518 10.1136/bmjopen-2018-024411 PMC6347902

[14] Bunchman, T. E., Maxvold, N. J. & Brophy, P. D. Pediatric convective hemofiltration: Normocarb replacement fluid and citrate anticoagulation. *Am. J. Kidney Dis.* **42**, 1248–1252 (2003). 14655197 10.1053/j.ajkd.2003.08.026

[15] Liet, J. M. et al. Regional citrate anticoagulation for pediatric CRRT using integrated citrate software and physiological sodium concentration solutions. *Pediatr. Nephrol.* **29**, 1625–1631 (2014). 24526097 10.1007/s00467-014-2770-2

[16] Persic, V. et al. Regional citrate anticoagulation for continuous renal replacement therapy in newborns and infants: Focus on citrate accumulation. *Artif. Organs* **44**, 497–503 (2020). 31851381 10.1111/aor.13619

[17] Elhanan, N., Skippen, P., Nuthall, G., Krahn, G. & Seear, M. Citrate anticoagulation in pediatric continuous venovenous hemofiltration. *Pediatr. Nephrol.* **19**, 208–212 (2004). 14669096 10.1007/s00467-003-1328-5

[18] Chadha, V., Garg, U., Warady, B. A. & Alon, U. S. Citrate clearance in children receiving continuous venovenous renal replacement therapy. *Pediatr. Nephrol.* **17**, 819–824 (2002). 12376810 10.1007/s00467-002-0963-6

[19] Fernández, S. N. et al. Citrate anticoagulation for CRRT in children: comparison with heparin. *Biomed. Res. Int.* **2014**, 786301 (2014). 25157369 10.1155/2014/786301 PMC4137493

[20] Rodriguez, K. et al. Regional citrate anticoagulation for continuous renal replacement therapy in pediatric patients with liver failure. *PLoS One* **12**, e0182134 (2017). 28792509 10.1371/journal.pone.0182134 PMC5549692

[21] Musielak, A. et al. Outcomes of Continuous Renal Replacement Therapy With Regional Citrate Anticoagulation in Small Children After Cardiac Surgery: Experience and Protocol From a Single Center. *Ther. Apher. Dial.* **20**, 639–644 (2016). 27786420 10.1111/1744-9987.12456

[22] Raymakers-Janssen, P. et al. Citrate versus heparin anticoagulation in continuous renal replacement therapy in small children. *Pediatr. Nephrol.* **32**, 1971–1978 (2017). 28578542 10.1007/s00467-017-3694-4 PMC5579151

[23] Kenet, G., Strauss, T., Kaplinsky, C. & Paret, G. Hemostasis and thrombosis in critically ill children. *Semin Thromb. Hemost.* **34**, 451–458 (2008). 18956285 10.1055/s-0028-1092875

[24] Bateman, S. T. et al. Anemia, blood loss, and blood transfusions in North American children in the intensive care unit. *Am. J. Respir. Crit. Care Med.* **178**, 26–33 (2008). 18420962 10.1164/rccm.200711-1637OC

[25] Sutherland, S. M. et al. Fluid overload and mortality in children receiving continuous renal replacement therapy: the prospective pediatric continuous renal replacement therapy registry. *Am. J. Kidney Dis.* **55**, 316–325 (2010). 20042260 10.1053/j.ajkd.2009.10.048

[26] Foland, J. A. et al. Fluid overload before continuous hemofiltration and survival in critically ill children: a retrospective analysis. *Crit. Care Med.* **32**, 1771–1776 (2004). 15286557 10.1097/01.ccm.0000132897.52737.49

[27] Cortina, G. et al. Mortality of Critically Ill Children Requiring Continuous Renal Replacement Therapy: Effect of Fluid Overload, Underlying Disease, and Timing of Initiation. *Pediatr. Crit. Care Med.* **20**, 314–322 (2019). 30431556 10.1097/PCC.0000000000001806

[28] Goldstein, B., Giroir, B. & Randolph, A. International pediatric sepsis consensus conference: definitions for sepsis and organ dysfunction in pediatrics. *Pediatr. Crit. Care Med.* **6**, 2–8 (2005). 15636651 10.1097/01.PCC.0000149131.72248.E6

[29] Schneider, A. G., Journois, D. & Rimmelé, T. Complications of regional citrate anticoagulation: accumulation or overload? *Crit. Care.* **21**, 281 (2017). 29151020 10.1186/s13054-017-1880-1 PMC5694623

[30] Valentine, S. L. et al. Consensus Recommendations for RBC Transfusion Practice in Critically Ill Children From the Pediatric Critical Care Transfusion and Anemia Expertise Initiative. *Pediatr. Crit. Care Med.* **19**, 884–898 (2018). 30180125 10.1097/PCC.0000000000001613 PMC6126913

[31] Buccione, E. et al. Continuous Renal Replacement Therapy in Critically Ill Children in the Pediatric Intensive Care Unit: A Retrospective Analysis of Real-Life Prescriptions, Complications, and Outcomes. *Front. Pediatr.* **9**, 696798 (2021). 34195164 10.3389/fped.2021.696798 PMC8236631

[32] Baldwin, I. Factors affecting circuit patency and filter ‘life’. *Contrib. Nephrol.* **156**, 178–184 (2007). 17464125 10.1159/000102081

[33] Davenport, A. & Tolwani, A. Citrate anticoagulation for continuous renal replacement therapy (CRRT) in patients with acute kidney injury admitted to the intensive care unit. *NDT* **6**, 439–447 (2009). 10.1093/ndtplus/sfp136 PMC4421325 25949376

[34] Betjes, M. G., Van Oosterom, D., Van Agteren, M. & Van De Wetering, J. Regional citrate versus heparin anticoagulation during venovenous hemofiltration in patients at low risk for bleeding: similar hemofilter survival but significantly less bleeding. *J. Nephrol.* **20**, 602–608 (2007). 17918147

[35] Zarbock, A. et al. Effect of Regional Citrate Anticoagulation vs Systemic Heparin Anticoagulation During Continuous Kidney Replacement Therapy on Dialysis Filter Life Span and Mortality Among Critically Ill Patients With Acute Kidney Injury: A Randomized Clinical Trial. *JAMA* **324**, 1629–1639 (2020). 33095849 10.1001/jama.2020.18618 PMC7585036

[36] Sık, G., Demirbuga, A., Annayev, A. & Citak, A. Regional citrate versus systemic heparin anticoagulation for continuous renal replacement therapy in critically ill children. *Int. J. Artif. Organs* **43**, 234–241 (2020). 31856634 10.1177/0391398819893382

[37] Askenazi, D. J. et al. Continuous renal replacement therapy for children ≤10 kg: a report from the prospective pediatric continuous renal replacement therapy registry. *J. Pediatr.* **162**, 587–592.e583 (2013). 23102589 10.1016/j.jpeds.2012.08.044 PMC5545826

[38] Rico, M. P. et al. Regional citrate anticoagulation for continuous renal replacement therapy in children. *Pediatr. Nephrol.* **32**, 703–711 (2017). 27896442 10.1007/s00467-016-3544-9

[39] Zaoral, T., Hladík, M., Zapletalová, J., Trávníček, B. & Gelnarová, E. Circuit Lifetime With Citrate Versus Heparin in Pediatric Continuous Venovenous Hemodialysis. *Pediatr. Crit. Care Med.* **17**, e399–e405 (2016). 27427878 10.1097/PCC.0000000000000860

[40] Cappoli, A. et al. A simplified protocol of regional citrate anticoagulation with phosphate-containing solutions in infants and children treated with continuous kidney replacement therapy. *Pediatr. Nephrol.* **38**, 3835–3844 (2023). 37222937 10.1007/s00467-023-05994-y

[41] Brophy, P. D. et al. Multi-centre evaluation of anticoagulation in patients receiving continuous renal replacement therapy (CRRT). *Nephrol. Dial. Transplant.* **20**, 1416–1421 (2005). 15855212 10.1093/ndt/gfh817

[42] Rodl, S. et al. One-year safe use of the Prismaflex HF20 (R) disposable set in infants in 220 renal replacement treatment sessions. *Intensive Care Med* **37**, 884–885 (2011). 21336778 10.1007/s00134-011-2147-y

[43] Ronco, C. et al. Continuous renal replacement therapy in neonates and small infants: development and first-in-human use of a miniaturised machine (CARPEDIEM). *Lancet* **383**, 1807–1813 (2014). 24856026 10.1016/S0140-6736(14)60799-6

[44] Köstekci, Y. E. et al. Evaluation of the efficacy and associated complications of regional citrate anticoagulation in neonates: experience from a fourth level neonatal intensive care unit. *Eur. J. Pediatr.* **182**, 4897–4908 (2023). 37597047 10.1007/s00431-023-05162-2

[45] Soltysiak, J. et al. Citrate anticoagulation for continuous renal replacement therapy in small children. *Pediatr. Nephrol.* **29**, 469–475 (2014). 24337319 10.1007/s00467-013-2690-6 PMC3913856

[46] Cortina, G., Daverio, M., Demirkol, D., Chanchlani, R. & Deep, A. Continuous renal replacement therapy in neonates and children: what does the pediatrician need to know? An overview from the Critical Care Nephrology Section of the European Society of Paediatric and Neonatal Intensive Care (ESPNIC). *Eur. J. Pediatr.* **183**, 529–541 (2024). 10.1007/s00431-023-05318-0 PMC10912166 37975941

[47] Miklaszewska, M. et al. Filter Size Not the Anticoagulation Method is the Decisive Factor in Continuous Renal Replacement Therapy Circuit Survival. *Kidney Blood Press Res* **42**, 327–337 (2017). 28578343 10.1159/000477609

[48] Martin, P. Y., Chevrolet, J. C., Suter, P. & Favre, H. Anticoagulation in patients treated by continuous venovenous hemofiltration: a retrospective study. *Am. J. Kidney Dis.* **24**, 806–812 (1994). 7977323 10.1016/s0272-6386(12)80675-5

[49] Huang, H. et al. Regional citrate anticoagulation for continuous renal replacement therapy in newborns. *Front Pediatr.* **11**, 1089849 (2023). 36969287 10.3389/fped.2023.1089849 PMC10030704

[50] Liet, J. M. et al. Semiautomated Regional Citrate Anticoagulation for Continuous Kidney Replacement Therapy: An Observational Study in Young Children. *Pediatr. Crit. Care Med.* **23**, e429–e433 (2022). 35583226 10.1097/PCC.0000000000002993

[51] Kozik-Jaromin, J., Nier, V., Heemann, U., Kreymann, B. & Böhler, J. Citrate pharmacokinetics and calcium levels during high-flux dialysis with regional citrate anticoagulation. *Nephrol. Dial. Transplant.* **24**, 2244–2251 (2009). 19196824 10.1093/ndt/gfp017 PMC2698091

[52] Jacobs, R. et al. Regional Citrate Anticoagulation in Continuous Renal Replacement Therapy: Is Metabolic Fear the Enemy of Logic? A Systematic Review and Meta-Analysis of Randomised Controlled Trials. *Life (Basel)* **17**, 1198 (2023). 10.3390/life13051198 PMC10221969 37240843

[53] Tolwani, A. J., Campbell, R. C., Schenk, M. B., Allon, M. & Warnock, D. G. Simplified citrate anticoagulation for continuous renal replacement therapy. *Kidney Int.* **60**, 370–374 (2001). 11422774 10.1046/j.1523-1755.2001.00809.x

[54] Poh, C. B. et al. Regional Citrate Anticoagulation for Continuous Renal Replacement Therapy - A Safe and Effective Low-Dose Protocol. *Nephrol. (Carlton)* **25**, 305–313 (2020). 10.1111/nep.13656 31469465

